# Supplementary material for: A genome-wide CRISPR screen identifies GRA38 as a key regulator of lipid homeostasis during Toxoplasma gondii adaptation to lipid-rich conditions
Source: Res Sq. 2025 Apr 18:rs.3.rs-6436164. Preprint. [Version 1] doi: 10.21203/rs.3.rs-6436164/v1 (PMC12047978; doi:10.21203/rs.3.rs-6436164/v1)
Supplement: 1 [file NIHPPRS6436164V1-supplement-1.pdf]

Table S1 Lipidomics Data

This table contains two sheets. The first sheet presents lipid species differentially abundant between human foreskin fibroblasts (HFFs) cultured in 1% versus 10% fetal bovine serum (FBS).

Each row represents a detected lipid species identified by its retention time and m/z value. Annotation refers to the putative lipid identity based on spectral matching or accurate mass and retention time. The assay column indicates the ionization mode used (negLipids = negative ion mode; posLipids = positive ion mode). Species denotes the ion adduct observed. InChIKey provides the standardized chemical identifier. Columns labeled HFFs-1/2/3 1% FBS and HFFs-1/2/3 10% FBS contain raw peak height values from three biological replicates cultured in 1% or 10% fetal bovine serum, respectively. The T-test column indicates the p-value from a two-tailed Student's t-test comparing lipid peak heights between 1% and 10% FBS. L2FC (1% vs 10%) reports the $\log_2$ fold change in lipid abundance under low serum (1% FBS) relative to high serum (10% FBS) conditions. Negative values indicate lower abundance in 1% FBS.

The second sheet contains *Toxoplasma* lipidomics data comparing wild-type, GRA38 knockout (GRA38KO), and complemented (GRA38COMP) parasites.

Table S2. CRISPR screen data identifying *Toxoplasma gondii* genes with differential fitness under low- and high-serum conditions.

This table contains results from a genome-wide CRISPR/Cas9 screen comparing Toxoplasma fitness in human foreskin fibroblasts (HFFs) cultured in 1% versus 10% fetal bovine serum (FBS). Each row represents a gene annotated with its ToxoDB ID, description, and additional functional information if available. Fitness scores from individual replicates (P4, P5, and P8; Exp1 and Exp2) under 1% and 10% FBS are provided, along with the calculated phenotype (10%–1%), p-values for depletion and enrichment, the number of high-quality sgRNAs supporting each phenotype, and adjusted p-values based on $\log_2$ fold change. Mean phenotype values and associated statistics across replicates are also included. This dataset was used to identify genes that influence parasite fitness in response to lipid availability. The raw count numbers for the number of reads for each sgRNA are also provided.

Table S3. Primers and antibodies used in this study.

This table lists all primers and antibodies used in the study. It includes primers used for Illumina sequencing of sgRNAs amplified from *Toxoplasma* genomic DNA, as well as oligonucleotides designed for gene complementation and endogenous tagging. Additional primers used for site-directed mutagenesis and other molecular biology applications are also included. Antibodies used in immunofluorescence and immunoblotting experiments are listed with corresponding details.

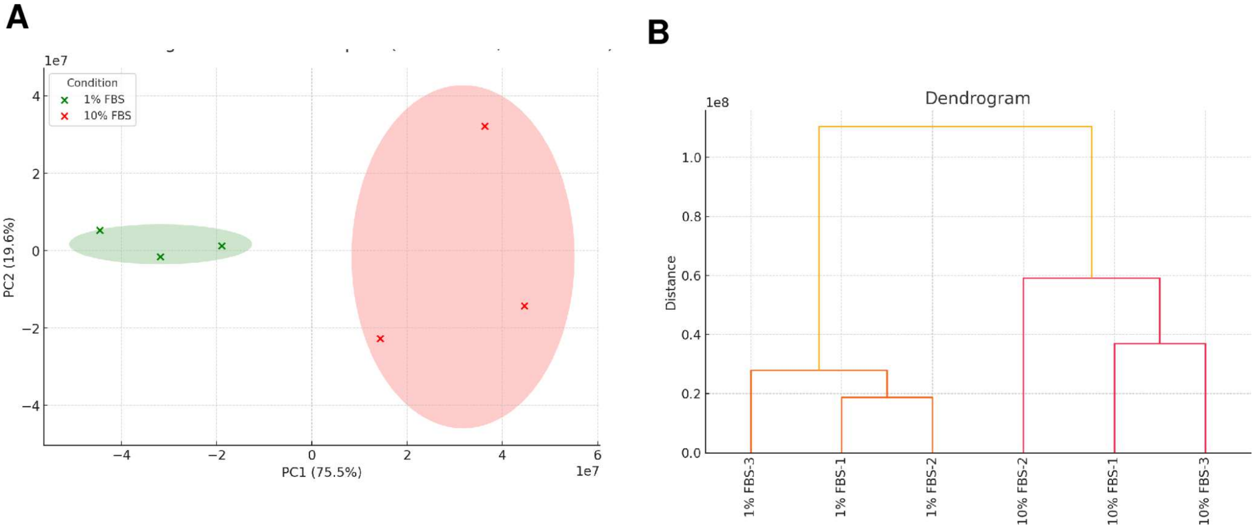

Figure S1. Metabolite profile differences in host cells grown under 1% and 10% FBS conditions.

(A) PCA plot displaying the separation of metabolite profiles between host cells grown in 1% FBS (green) and 10% FBS (red). Each point represents an individual sample, with the principal components capturing the variation in the dataset. The clear separation of the two groups indicates distinct metabolomic changes due to the different FBS concentrations. **(B)** Hierarchical clustering dendrogram showing the relationship among samples based on their metabolite profiles. Samples grown in 1% FBS cluster separately from those grown in 10% FBS, supporting the PCA findings of metabolic divergence under the two conditions.

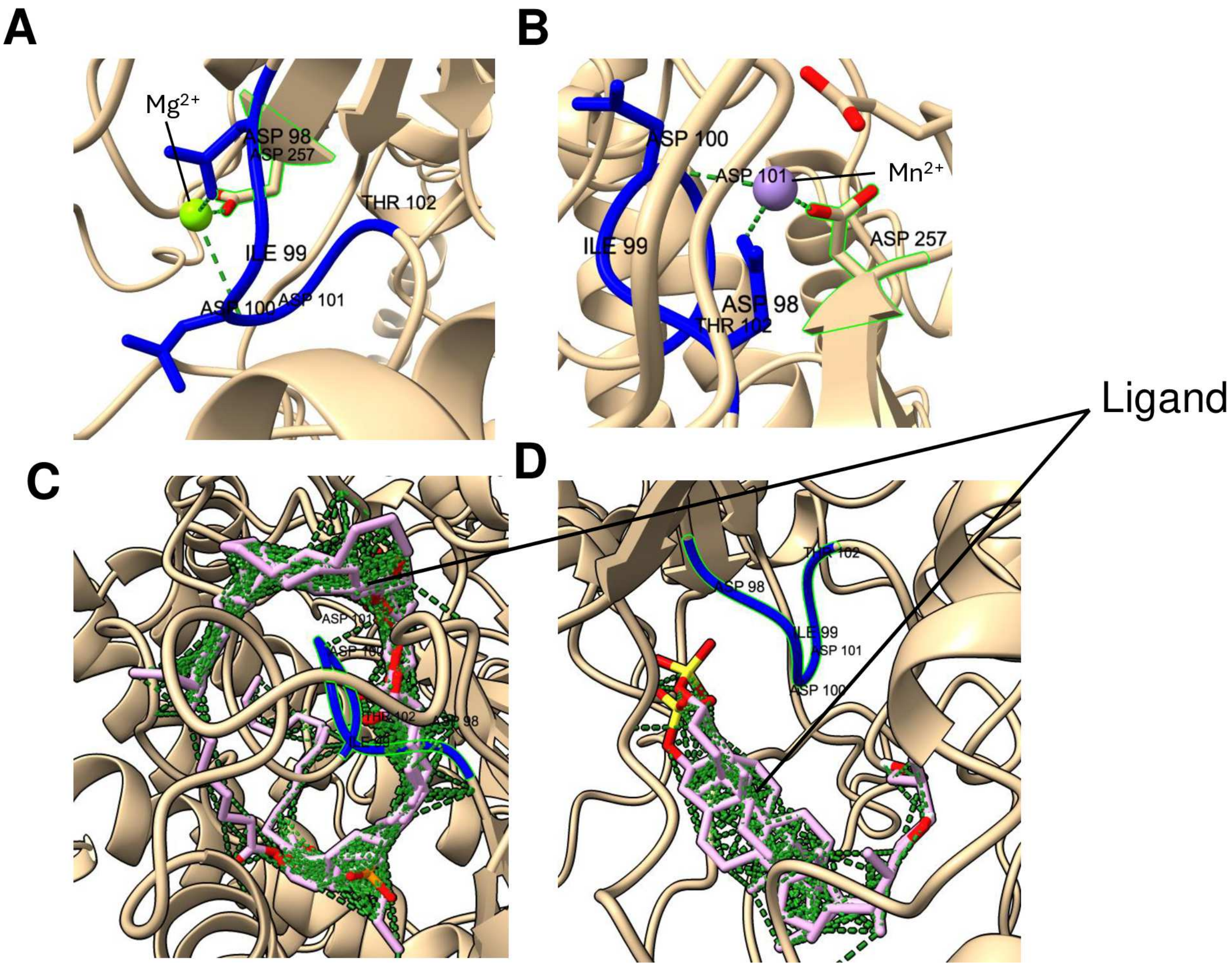

Figure S2. **Structural insights into GRA39 and its DxDxT/V catalytic motif.**

(A) Structural view of the GRA39 DxDxT/V motif residues bound to magnesium, with polar contacts indicated by green dashes. **(B)** Close-up view of the key residue interactions in the active catalytic site with manganese bound, highlighting the structural rearrangement. **(C)** Docking of PA to GRA39 by AutoDockVina, showing direct interactions with the DxDxT/V catalytic motif within the GRA39 binding pocket. **(D)** Docking of cholesterol as a non-substrate lipid control.

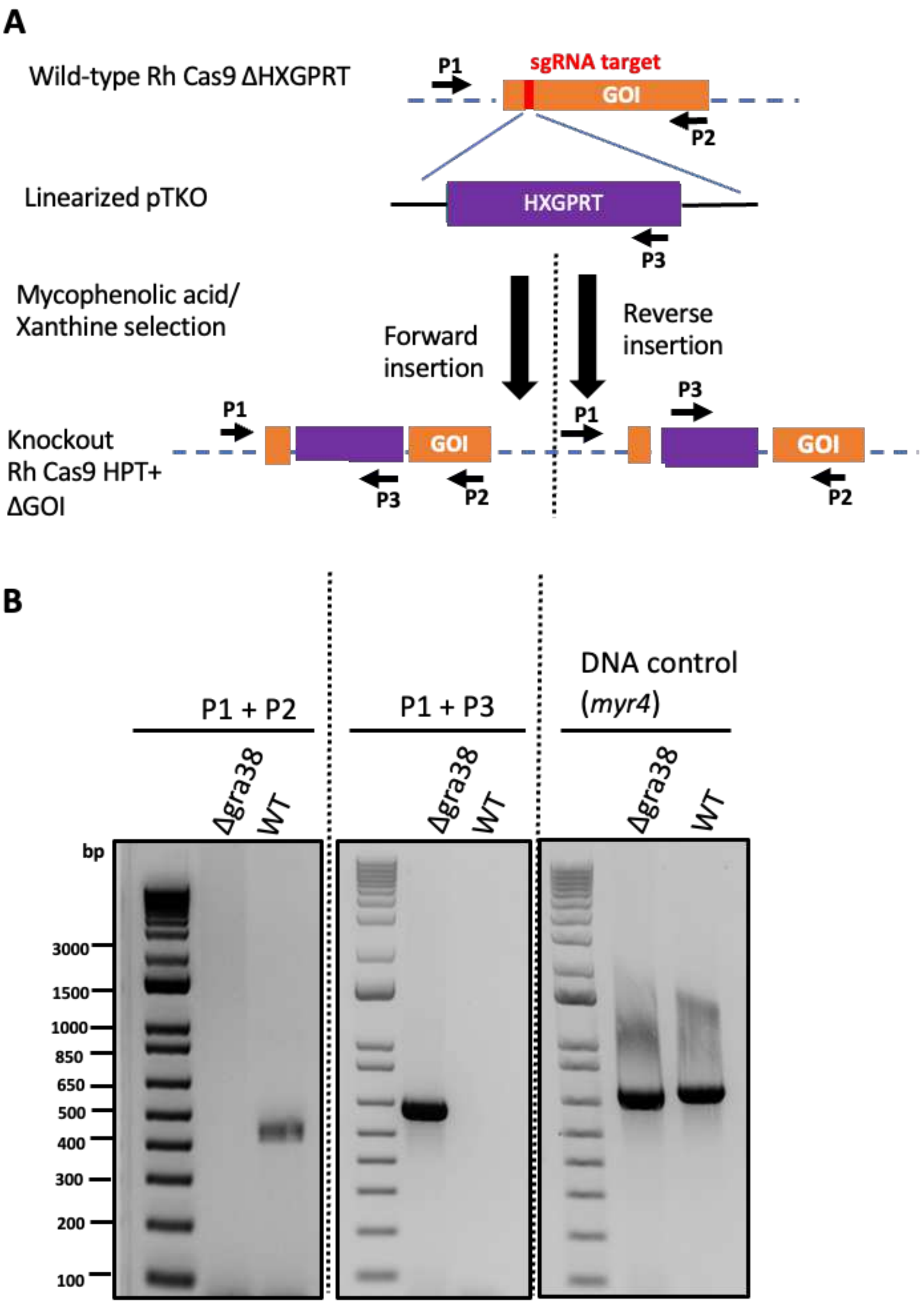

Figure S3. Generation of the *GRA38* knockout parasite strain.

(A) Schematic diagram illustrating the strategy used to delete *GRA38* in the type 1 (RH)

strain. The CRISPR/Cas9-targeting site is highlighted by a red box. A linearized pTKO plasmid carrying an HXGPRT selection cassette served as the repair template; selection was performed using mycophenolic acid and xanthine. **(B)** Confirmation of gene-of-interest (GOI) disruption was performed using primers P1 and P2, which amplify a region within the GOI, with MYR4 serving as a PCR control. Successful insertion of the repair template was verified using primers P1 and P3.

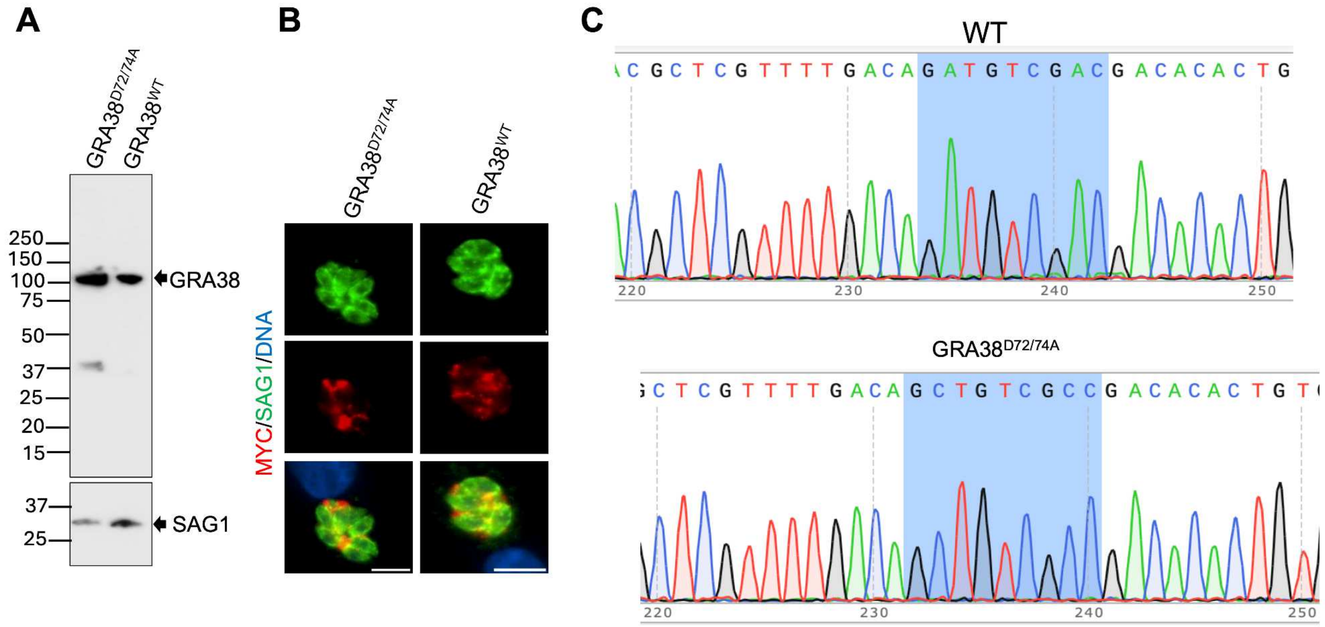

Figure S4. Confirmation of complemented strains.

(A) Western blot analysis showing complementation of the *Δgra38* knockout in the type 1 (RH) background with either full-length or inactive GRA38, each expressing a C-terminal MYC tag. The GRA38 protein (117.82 kDa) was detected using an anti-MYC antibody. **(B)** Immunofluorescence assay showing the localization of GRA38 (in red) in complemented *Δgra38* parasites. Scale bar = 6 μM. **(C)** Confirmation of DxDxT/V motif mutations. Genomic DNA was extracted from WT GRA38 or GRA38$^{D72/74A}$ parasites,

followed by PCR amplification. Sanger sequencing of the PCR amplicon confirmed mutation of the DxDxT/V motif into AxAxT/V.

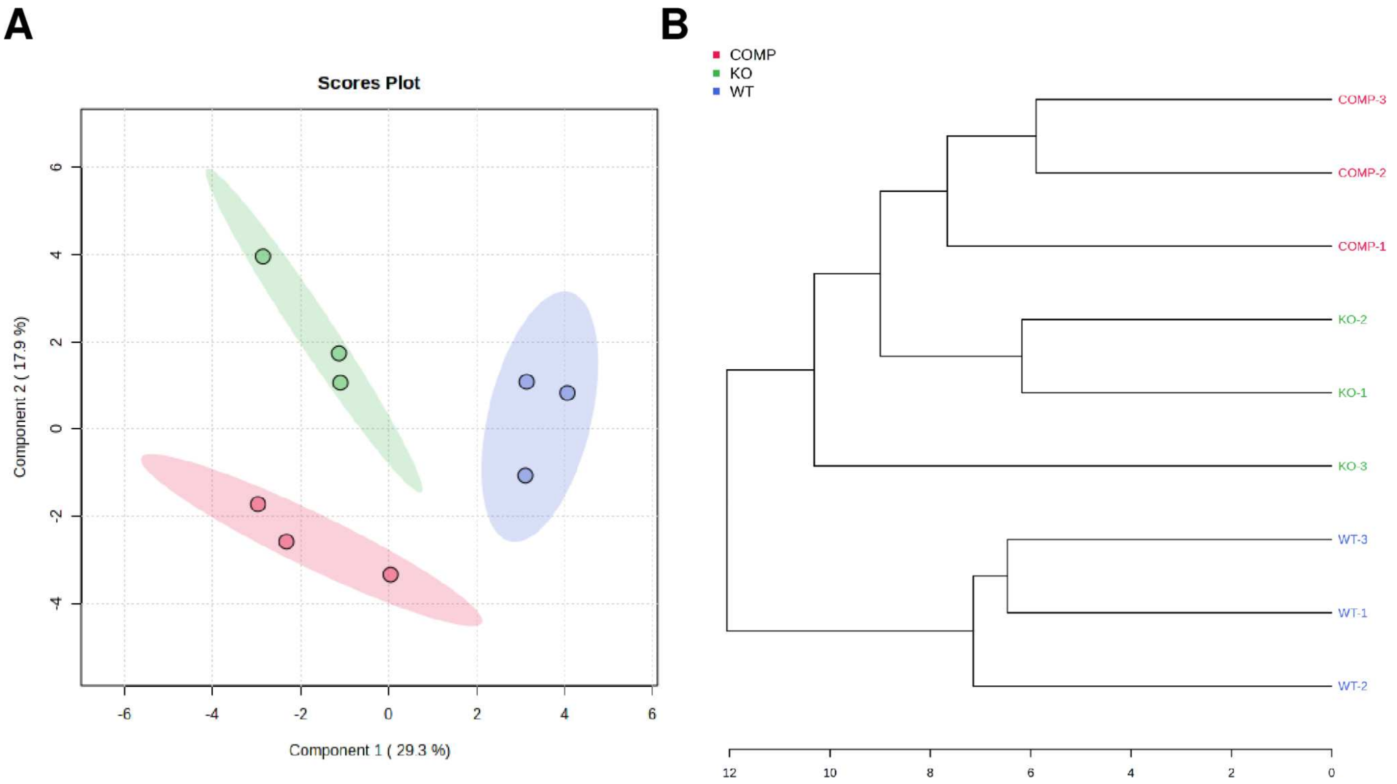

Figure S5. Principal component analysis and hierarchical clustering of WT, Δ*gra38*, and complemented parasite strains. (A) PCA derived from the combined data of WT (blue), KO (*Δgra38* knockout, green), and COMP ($GRA38^{WT}$, red). Each point represents a biological replicate. **(B)** Hierarchical clustering dendrogram showing the relationships among the three experimental groups based on their metabolic profiles. The distance between clusters indicates the similarity among samples.
